# 284 How Clean is Clean? Mitigation Efforts of Ice/water Dispensers Linked to a Mycobacterium chelonae Outbreak at a Pediatric Hospital

**DOI:** 10.1017/ash.2026.10735

**Published:** 2026-06-23

**Authors:** Alyssa Olson, Jean Barth, Jenna Rasmusson, Nicole Kriewall, Cory Kudrna, Todd Sheley, Tyler Kramer, Jason Buckmeier, John OHoro

**Affiliations:** 1 Mayo Clinic; 2 Mayo Clinic College of Medicine

## Abstract

**Background:** We had a large-scale measles exposure event affecting 626 patients. Management of exposed patients was urgent, involving quarantine, post-exposure prophylaxis (PEP), and symptom monitoring on a case-by-case basis. Cases were initially tracked and managed using a combination of multiple spreadsheets amongst stakeholders as well as lists within the electronic health record (EHR). Using shared spreadsheets and lists created significant challenges with maintaining and editing multiple copies and updating workbooks simultaneously. **Methods:** Infection Prevention and Control (IPAC) worked with primary care personnel and EHR developers to create a shared registry that would prioritize communication and follow-up for exposed patients based on age and immunity. Patients are initially added to the shared registry within the EHR by reviewing non-registry reports and tagging patients, identified using exposure timeframes that overlapped with the index measles cases, in a manner that transferred them to the shared registry. From this registry, measles, mumps, and rubella vaccine status can be confirmed, immune status can be identified, and criteria for PEP assigned. High-risk patients are then contacted with recommendations from IPAC and primary care. Communication and follow-up are tracked directly within the shared registry to ensure appropriate patient management. The exposure management registry is further leveraged throughout the measles outbreak timeframe by allowing real-time monitoring of patients that require additional precautions or guidance related to their exposure. **Result:** The shared registry enhanced documentation accuracy and transparency between stakeholders. It led to availability of real-time status of exposed patients for the duration of the measles outbreak timeframe. Overall, the registry enabled time-sensitive patient communication to all exposed patients and rapid identification of those who needed PEP. Communication could happen rapidly and seamlessly between care teams within the organization, and the necessary information could be shared quickly with public health authorities as required. **Conclusion:** Large scale exposures are a challenge to manage and ensure appropriate action and communication occurs. Errors can be prevented by having a single shared exposure management registry. A registry enables care teams to quickly identify and prioritize patients for measles follow-up, communication, PEP, and ongoing management across outbreak timeframes. This process reduces both time and operational burdens of exposure follow-up tracking outside of the EHR.

